# Pain sensitivity and quality of life of patients with burning mouth syndrome: a preliminary study in a Chinese population

**DOI:** 10.1186/s12903-023-03689-2

**Published:** 2023-12-01

**Authors:** Hongsen Zhao, Shujun Ran, Kang Gan, Yajing Du, Wenlu Li

**Affiliations:** 1 Department of Stomatology, The First Affiliated Hospital of Zhengzhou University, No. 1 Jianshe East Road, Zhengzhou City, 450052 China; 2 National Clinical Research Center for Oral Diseases, Shanghai, China

**Keywords:** Burning mouth syndrome, Pain sensitivity, Oral health-related quality of life, Pain Sensitivity Questionnaire

## Abstract

**Background:**

Burning mouth syndrome (BMS) is an oral-facial pain disorder involving the central and peripheral nervous systems, but the evidence for altered pain sensitivity remains inconclusive. The aim of this study was to investigate pain sensitivity and oral health-related quality of life (OHRQoL) in patients with BMS and to assess the relationship between them.

**Methods:**

Fifty Chinese patients with BMS (57.82 ± 11.2 years) and fifty age- and gender-matched healthy subjects (55.64 ± 10.1 years) participated in the study. The Pain Sensitivity Questionnaire (PSQ) was used to assess participants’ pain sensitivity. The Oral Health Impact Profile (OHIP-14) was used to evaluate participants’ OHRQoL.

**Results:**

The PSQ total score (p = 0.009), the PSQ minor score (p = 0.003) and the OHIP-14 score (p<0.05) of patients with BMS were significantly higher than those of the healthy subjects. Simple linear regression showed that the PSQ minor score was significantly associated with the OHIP-14 score in patients with BMS (β = 0.338, p = 0.016).

**Conclusion:**

Patients with BMS have higher pain sensitivity than healthy subjects. Reducing pain sensitivity might help to improve the quality of life of patients with BMS.

## Introduction

Burning mouth syndrome (BMS) is a chronic oral mucosal pain that often causes a persistent burning sensation and sensory dysfunction with a lack of clinical and laboratory findings [1, 2]. The International Association for the Study of Pain (IASP) defines BMS as “chronic burning pain in the mouth for which local or systemic causes cannot be determined” [3]. The global prevalence of the disease is approximately 4% and varies widely due to differences in BMS definitions and inclusion criteria. In a population-based study, the prevalence of BMS in residents aged 17–92 years in Shanghai, China, was estimated to be 1.38% [4]. The prevalence is highest in postmenopausal women and older women [5]. The burning sensation typically affects the tongue (especially the tip and lateral margins), lips, and soft and hard palate and is often accompanied by xerostomia, taste disturbances, or food allergies [6, 7]. The pathogenesis of BMS is unknown, and neuroendocrine dysfunction may precipitate or exacerbate the disease [8]. Sevrin et al., using the Douleur Neuropathique 4 questionnaire, suggested that 30-60% of BMS patients have neuropathic pain components [9]. Other questionnaire-based studies also supported the view that BMS is a clinical manifestation of a neurological disease [10, 11]. Earlier tongue biopsy analysis showed that the density of intraepidermal and epithelial nerve fibers in BMS patients was significantly lower than that in healthy subjects [12–14]. In fact, neurophysiological, psychophysical and functional imaging studies have shown pathophysiological alterations at different levels of the neural axis, and BMS is currently believed to be neuropathic pain affecting the central and peripheral nervous systems [15]. Studying the clinical features and sensory changes associated with BMS might help in BMS management and treatment.

Above-average pain sensitivity may be a risk factor for progression to chronic pain [16]. Studies have found altered pain sensitivity in patients with BMS [17, 18]. Sensory changes associated with chronic pain are assessed in a standardized manner by quantitative sensory testing (QST), which is used in BMS studies. There were large individual differences in pain sensitivity, and inconsistent conclusions were obtained due to different inclusion criteria, experimental pain stimulation methods, and evaluation times [19–27]. Previous studies have focused on the determination of local experimental pain thresholds. When relying on pain thresholds, pain perception is measured only at a perceptible level, and the perception of suprathreshold pain intensity more similar to clinical pain is largely an independent dimension of pain sensitivity. The intensity level of the imagined pain may be more closely related to the intensity level of clinical pain [28, 29]. The Pain Sensitivity Questionnaire (PSQ) is a self-rating instrument, taking a few minutes to complete, that asks respondents to imagine themselves in painful situations that are commonly experienced, and to rate the pain they feel they would experience [30]. The questionnaire is simple, requiring no equipment or extensive training, inducing no anxiety in subjects or patients at the prospect of an imminent ‘pain test’. It has been used in several chronic pain studies and is considered an alternative to experimental pain testing [31–33]. To date, there have been no reports of the PSQ being used in BMS.

The impact of BMS on patients’ lives is complex and multifaceted, and a persistent burning sensation adversely affects patients’ quality of life [34, 35]. Health-related quality of life (HRQoL) is a core area of pain management interventions. HRQoL is founded primarily on functionalism and reflects the currently recognized importance of assessing and treating not only the biological but also the psychological and social factors contributing to a patient’s chronic pain condition [36, 37]. Poor oral health-related quality of life (OHRQoL), in turn, may affect a patient’s experience of pain. In this study, we intended to investigate pain sensitivity using the Pain Sensitivity Questionnaire (PSQ) in BMS patients in China and explore the association between pain sensitivity and OHRQoL. We hypothesized that patients with BMS present higher pain sensitivity than healthy subjects, and higher level of pain sensitivity might deteriorate OHRQoL in BMS patients.

## Methods

### Participants

This cross-sectional study was conducted from January 2021 to January 2023. The study sample consisted of patients diagnosed with BMS who were attending the dental clinic at The First Affiliated Hospital of Zhengzhou University. The study protocol followed the principles established in the Declaration of Helsinki and was approved by the Medical Research Ethics Committee of the First Affiliated Hospital of Zhengzhou University(2020-KY-457). All patients were volunteers, provided informed consent to participate and received no remuneration.

The inclusion criteria for the diagnosis of BMS in this study were as follows [38]: (1) patients complained of burning pain in the mouth; (2) patients may have had other subjective symptoms (such as xerostomia, oral paresthesia and taste disorders); (3) no obvious oral lesions were observed; and (4) there was a lack of evidence for a specific etiology that may cause the oral burning sensation, such as trigeminal neuralgia, diabetes, malnutrition, and connective tissue disease. The control group was healthy subjects who underwent dental condition examinations in the dental clinic, did not receive treatment, had no history of chronic pain syndrome, and did not have oral and maxillofacial pain caused by local diseases.

The target sample size was set by fixing a power test value (1-Beta) no less than 80% associated with a significance of no more than 5% and an effect size value of 0.6. The calculations were computed using G*power software (v 3.1.9). Finally, 50 BMS patients and 50 healthy subjects were enrolled in this study.

### Assessment of pain sensitivity

Patients with BMS and healthy subjects were assessed by the Pain Sensitivity Questionnaire (PSQ), a clinically useful and effective self-assessment method for pain perception in patients with chronic pain based on pain intensity scores in everyday life situations. The PSQ consists of 17 items that are scored on a scale from 0 (no pain at all) to 10 (the worst pain imaginable) on a numerical rating scale (NRS). The total PSQ score consists of two subscales (PSQ minor and PSQ moderate) and is the average score for all but three nonpain items. Mean scores of < 4, 4–6, and > 6 were considered mild, moderate, and severe pain sensitivity, respectively. Although the correlation between PSQ minor scores and experimental pain intensity ratings was better than that between total PSQ and PSQ moderate scores, this study collected both total PSQ scores and PSQ minor scores. The Mandarin version of the PSQ has been validated in Chinese groups [39].

### Determination of OHRQoL

The Oral Health Impact Scale (OHIP-14) is the most widely used instrument to assess OHRQoL. The OHIP-14 comprises 14 items that describe 7 domains: functional limitations, physical pain, psychological discomfort, physical disability, mental disability, social disability, and social handicap. Items are scored on a 5-point Likert scale as follows: 0 = none; 1 = rare; 2 = sometimes; 3 = often and 4 = very often. The OHIP-14 total scores, ranging from 0 to 56, were obtained by summing the scores for all 14 items, with higher OHIP-14 scores indicating worse OHRQoL. The Chinese version of the OHIP-14 has been validated in Chinese groups [40].

### Data analysis

All analyses were performed using SPSS software version 22.0 (SPSS Corporation, Chicago, Illinois, USA). A descriptive study was performed on each variable. The two groups were compared using Student’s t test, chi-square test, or Mann‒Whitney U test. Simple linear regression was used to determine the correlation between the PSQ score and the OHIP-14 score. P < 0.05 was accepted as statistically significant. The R^2^ value represents the explanatory power of the regression model.

## Results

### Clinical features

The demographic and clinical characteristics of the subjects are shown in Table 1. The mean age of all patients was 56.7 ± 10.7 years, and the sample included 91 (91%) women and 9 (9%) men. Of the 91 female patients, 70 (76.9%) were menopausal. The patients with BMS consisted of 5 males and 45 females, aged 36 ~ 84 years old, with an average age of 57.82 ± 11.2 years.

**Table 1:** Demographic and clinical characteristics of patients with BMS and healthy controls

		BMS	Controls	p-values
**Age(years)**		57.82 ± 11.2	55.64 ± 10.1	0.314^a^
	Range	36 ~ 84	33 ~ 79	
**Sex**				
	Male	5 (10%)	4 (8%)	0.727^b^
	Female	45 (90%)	46 (92%)	
**Menopause**				
	Yes	37 (74%)	33 (66%)	0.383^b^
	No	13 (26%)	17 (34%)	
**Pain Duration(months)**		9.62 ± 8.64	/	
	Range(month)	1 ~ 48		
**Pain location**				
	Tongue	47 (94%)	/	
	Hard palate	13 (26%)	/	
	Lip	9 (18%)	/	
	Buccal mucosa	6 (12%)	/	
**Oral complaints**				
	Oral burning sensation	50 (100%)	/	
	Xerostomia	37 (74%)	/	
	Taste disturbance	25 (50%)	/	

Abbreviation: BMS, burning mouth syndrome. ^a^*t*-test, ^b^Pearson’s chi-squared test

The healthy subjects consisted of 4 males and 46 females, aged 33 ~ 79 years old, with an average age of 55.64 ± 10.1 years. The duration of BMS oral symptoms had a wide range, from a minimum of 1 month to a maximum of 48 months. The mean duration of disease was 9.62 ± 8.64 months. Most patients reported symptoms on the tongue (n = 47, 94%), followed by the anterior hard palate (n = 13, 26%) and labial mucosa (n = 9, 18%). All fifty patients (100%) had the typical burning mouth pain. Thirty-seven (74%) patients reported dry mouth, and 25 (42%) patients reported dysgeusia. Nineteen patients (38%) reported the classic symptom triad of BMS, including burning mouth symptoms, taste disturbances, and xerostomia. Thirty-six patients (72%) reported symptoms persisting throughout the day. Eleven patients (22%) reported asymptomatic waking in the morning, increasing gradually throughout the day and peaking in the evening. Three patients (6%) reported intermittent symptoms on some days.

### Pain sensitivity

PSQ subscale scores of patients with BMS and healthy controls are shown in Table 2. The total PSQ score ranged from 2.5 to 8.5 in the patients with BMS and 1.64 to 6.5 in healthy subjects. The total PSQ score of patients with BMS was significantly higher than that of the healthy subjects (5.05 ± 1.23 vs. 4.45 ± 0.98, p = 0.009). In the subscale analysis, the PSQ minor score of patients with BMS was significantly higher than that of the healthy subjects (4.20 ± 1.27 vs. 3.52 ± 0.92, p = 0.003), while there was no significant difference in the PSQ moderate score between the two groups (5.91 ± 1.46 vs. 5.38 ± 1.22, p = 0.058).

**Table 2:** PSQ subscale scores of patients with BMS and healthy controls

Variable	BMS	Healthy control	p-values^a^
**PSQ-Total**	5.05 ± 1.25	4.45 ± 0.99	0.009^*^
Median	4.79	4.54	
25% percentile	4.27	3.64	
75% percentile	5.45	5.29	
**PSQ-Minor**	4.20 ± 1.28	3.52 ± 0.93	0.003^*^
Median	4.21	3.57	
25% percentile	3.57	3.25	
75% percentile	4.61	4	
**PSQ-Moderate**	5.91 ± 1.48	5.39 ± 1.24	0.058
Median	4.21	3.57	
25% percentile	3.57	3.25	
75% percentile	4.61	4	

Abbreviation: BMS, burning mouth syndrome. ^a^*t*-test, ^*^p < 0.05

### Quality of life related to oral health

The OHIP-14 scores of patients with BMS and healthy controls are shown in Table 3. The OHIP-14 score was significantly higher in patients with BMS than in the healthy control group (24.56 ± 10.24 vs. 8.9 ± 6.46, p < 0.05). Compared with the healthy subjects, patients with BMS had significant differences in all domains. Among the OHIP-14 items, the third item, ‘painful aching in mouth’, in the physical pain domain had the highest score (3.06 ± 1.01), followed by the ninth item, ‘difficult to relax’, in the psychological disability domain (2.8 ± 1.16); the sixth item, “felt tense”, in the psychological discomfort domain (2.58 ± 1.31); and the fourth item, “uncomfortable eating anything”, in the physical pain domain (2.48 ± 1.22).

**Table 3:** OHIP-14 scores of patients with BMS and healthy controls

		BMS	Controls	p-values^a^
		Mean ± SD	Mean ± SD	
**Total**		24.56 ± 10.34	8.9 ± 6.52	0.000^*^
**Functional limitation**				
1.	Trouble pronouncing words	1.08 ± 1.21	0.44 ± 0.61	0.001^*^
2.	Sense of taste worse	1.32 ± 1.22	0.58 ± 0.57	0.000^*^
**Physical pain**				
3.	Painful aching in mouth	3.06 ± 1.01	1.58 ± 0.88	0.000^*^
4.	Uncomfortable eating anything	2.48 ± 1.22	1.3 ± 0.95	0.000^*^
**Psychological discomfort**				
5.	Been self-conscious	1.7 ± 1.25	0.28 ± 0.54	0.000^*^
6.	Felt tense	2.58 ± 1.31	1.38 ± 1.05	0.000^*^
**Physical disability**				
7.	Diet has been unsatisfactory	1.5 ± 1.27	0.64 ± 0.83	0.000^*^
8.	Had to interrupt meals	0.72 ± 0.86	0.24 ± 0.52	0.001^*^
**Psychological disability**				
9.	Difficult to relax	2.8 ± 1.16	0.86 ± 0.86	0.000^*^
10.	Been embarrassed	1.3 ± 1.37	0.26 ± 0.44	0.000^*^
**Social disability**				
11.	Been irritable with others	1.7 ± 1.37	0.5 ± 0.54	0.000^*^
12.	Difficulty doing usual jobs	1.2 ± 1.41	0.16 ± 0.37	0.000^*^
**Handicap**				
13.	Felt life is less satisfying	2.34 ± 1.26	0.58 ± 0.76	0.000^*^
14.	Totally unable to function	0.78 ± 1.11	0.12 ± 0.39	0.000^*^

Abbreviation: BMS, burning mouth syndrome. ^a^*t*-test, ^*^p < 0.05

### Correlation between OHIP-14 score and PSQ in patients with BMS

Simple linear regression analysis was performed on the OHIP-14 score and PSQ score of patients with BMS. There was no significant correlation between total PSQ scores and OHIP-14 scores (β = 0.214, p = 0.135), but PSQ minor scores were significantly correlated with OHIP-14 scores (β = 0.338, p = 0.016 < 0.05) (Table 4).

**Table 4:** Relationships between OHIP-14 scores and PSQ subscale scores in patients with BMS

Variable	OHIP-14					
	B	SE	β	t	p	R^2^
PSQ-total	1.78	1.170	0.214	1.521	0.135	0.046
PSQ-minor	2.735	1.098	0.338	2.491	0.016^*^	0.114
PSQ-moderate	0.481	1.008	0.069	0.477	0.635	0.005

The data were analyzed by the simple linear regression. ^*^p < 0.05

## Discussion

The main complaint reported by patients affected by BMS is pain. This is, therefore, the primary element to consider in any BMS diagnosis [41]. In a biopsychosocial context, the subjective experience of pain in patients with BMS is influenced by psychological, social, and cultural dimensions. It has been reported previously that pain sensitivity varies greatly between individuals and ethnicities [42]. Our study in Chinese patients found that patients with BMS had higher PSQ minor scores and that the scores were associated with OHIP-14 scores, i.e., higher pain sensitivity was associated with poorer oral-related quality of life in patients with BMS. This has not been reported in previous studies.

Pain sensitivity had two dimensions: pain threshold and pain intensity rating [29, 30]. The Pain Sensitivity Questionnaire (PSQ) developed by Ruscheweyh et al. had a high correlation with experimental pain intensity scores, although this correlation was not absolute [29]. Pain sensitivity here can be described as “general pain sensitivity”, representing the average pain level of the subject at a particular location and time point, not specifically to the field of pain disorders or local sensitivity of the mouth [30]. In our study, PSQ minor scores, consisting of mild pain scenario items, increased in the BMS patients, while there were no significant differences in total PSQ and PSQ moderate scores compared to controls. This may indicate increased sensitivity to mild pain in patients with BMS.

Unlike our study, earlier studies assessed changes in pain perception in BMS patients by using quantitative sensory testing (QST) to detect pain thresholds. Those studies found that the warmth detection threshold (WDT) [21, 22, 24–26] and cold detection threshold (CDT) of patients with BMS were significantly increased at the tongue [21, 25], indicating that both thermal function and sensitivity to thermal nonnoxious stimuli were reduced. Kaplan et al. found that there were no differences in WDT, CDT, cold pain threshold (CPT), or heat pain threshold (HPT), while age correlated with an increase in WDT but not BMS [27]. In addition, it was reported that the sensory function of BMS patients was reduced, with the mechanical pain threshold (MPT) and mechanical detection threshold (MDT) increased at the tongue [24]. Additionally, Honda et al. evaluated the mechanical sensitivity of the tongue using the tactile detection threshold (TDT) and filament sting detection threshold (FPT) and found no significant difference [20]. Conflicting results in the experimental assessment of pain sensitivity may be due to the differences in psychophysical techniques, devices applied, and clinical features of the subjects. The pain threshold only reflected the perceived level of pain perception, but the pain intensity rating for suprathreshold stimulation was more similar to clinical pain, which by definition is over the threshold [23]. In fact, experimental pain intensity ratings showed greater clinical relevance than experimental pain thresholds. It was previously reported that PSQ scores had no correlation with pain thresholds but had a strong association with pain intensity ratings [30]. Previous literature has focused on changes in pain perception in localized areas of the oral and maxillofacial area, where pain sensitivity in the head region and outside the head was not always consistent and was not representative of the patient’s general pain sensitivity. These factors might explain the difference between our findings and previous results.

As recommended by IMMPCT, quality of life assessment can help in the management of chronic pain [36]. Previously, scholars suggested that the OHIP-14 was an essential tool for assessing OHRQoL and should always be included in the evaluation of patients with BMS to better understand the bidirectional correlation between pain and self-perceived oral health [41]. Consistent with previous studies, oral health-related quality of life was significantly reduced in patients with BMS. We included more Chinese BMS patients than previous studies [34]. Due to differences in measurement tools, it was difficult to directly compare the results of BMS patients in our study with those of previous studies. Compared to previous studies using the same tool, OHIP-14, our BMS patients had lower OHIP-14 scores [43]. This variation may result from different exclusion criteria for patients, as well as differences in ethnicity and sociocultural background. In this study, high scores were obtained in the domains of physical pain, psychological disability, and psychological discomfort, suggesting that pain was the primary complaint of patients with BMS and was often accompanied by psychological problems.

In our study, simple linear regression analysis was used to find a significant correlation between pain sensitivity and quality of life in patients with BMS. A higher level of pain sensitivity worsened the OHRQoL of BMS patients, which had not been previously reported. Similar reports have been made in other types of diseases. A correlation between pain threshold and quality of life has been reported in patients with fibromyalgia [44]. Reducing pain sensitivity in patients with androgen deficiency can help improve their quality of life [45]. It has been accepted that chronic pain patients exhibiting high pain sensitivity responded less well to treatment than those with lower pain sensitivity [30]. Lowering the pain sensitivity in patients with BMS might contribute to increasing the effectiveness of treatment. Considering that patients with elevated pain sensitivity present a predisposition to develop chronic pain disorders [16], reducing pain sensitivity might help to prevent the recurrence of BMS. In summary, reducing pain sensitivity might help improve mental health, health function, and quality of life in patients with BMS.

This study presented some limitations and required a degree of caution in interpreting our findings. This was a cross-sectional survey conducted at a single point in time, with a limited sample size and no follow-up surveys. In this study, although we reported a correlation between the two, we could not prove whether patients with increased pain sensitivity were predisposed to BMS or whether long-term chronic pain stimulation during the course of BMS resulted in increased pain sensitivity, and longitudinal studies are needed to further clarify the correlation. The effectiveness of this research is, therefore, exploratory and should be interpreted with care on account of the small size of the sample. Future studies with larger samples of BMS patients are warranted to exploring the relationship between pain sensitivity and BMS, as well as potential interventions to reduce pain sensitivity in BMS patients.

## Conclusion

The quality of life of patients with BMS was significantly reduced. Compared with the healthy subjects, patients with BMS presented significantly heightened pain sensitivity. There was a correlation between the two. Reducing the pain sensitivity of BMS patients may be a potential way to improve the quality of life of patients with BMS, which is worthy of further exploration.

## Data Availability

The datasets used and analyzed during the current study are available from the corresponding author on reasonable request.

## Abbreviations

BMS: Burning mouth syndrome
CDT: Cold detection threshold
CPT: Cold pain threshold
FPT: Filament sting detection threshold
HPT: Heat pain threshold
IASP: International Association for the Study of Pain
MDT: Mechanical detection threshold
MPT: Mechanical pain threshold
OHIP-14: Oral health impact profile
OHRQoL: Oral health-related quality of life
PSQ: Pain Sensitivity Questionnaire
QST: Quantitative Sensory Testing
SD: Standard deviation
SE: Standard error
TDT: Tactile detection threshold
WDT: Warmth detection threshold
